# The Genetic Germline Background of Single and Multiple Primary Melanomas

**DOI:** 10.3389/fmolb.2020.555630

**Published:** 2021-03-05

**Authors:** Simona De Summa, Antonia Lasorella, Sabino Strippoli, Giuseppe Giudice, Gabriella Guida, Rossella Elia, Eleonora Nacchiero, Amalia Azzariti, Nicola Silvestris, Michele Guida, Stefania Guida, Stefania Tommasi, Rosamaria Pinto

**Affiliations:** ^1^Pharmacogenetics and Molecular Diagnostic Unit, IRCCS Istituto Tumori “Giovanni Paolo II”, Bari, Italy; ^2^Medical Oncology Unit, IRCCS Istituto Tumori “Giovanni Paolo II”, Bari, Italy; ^3^Department of Plastic and Reconstructive Surgery, University of Bari, Bari, Italy; ^4^Department of Basic Medical Science and Sense Organs, University of Bari, Bari, Italy; ^5^Pharmacology Laboratory IRCCS Istituto Tumori “Giovanni Paolo II”, Bari, Italy; ^6^Medical Oncology Unit IRCCS Istituto Tumori “Giovanni Paolo II”, Bari, Italy; ^7^Biomedical Sciences and Human Oncology (DIMO), University of Bari “Aldo Moro”, Bari, Italy; ^8^Dermatology Unit, Department of Surgical, Medical, Dental and Morphological Sciences Related to Transplant, Oncology and Regenerative Medicine, University of Modena and Reggio Emilia, Modena, Italy

**Keywords:** targeted next generation sequencing, genetics, single primary melanoma, germline mutations, multiple primary melanomas

## Abstract

**Background:** Melanoma has a complex molecular background and multiple genes are involved in its development and progression. The advent of next generation sequencing platforms has enabled the evaluation of multiple genes at a time, thus unraveling new insights into the genetics of melanoma. We investigated a set of germline mutations able to discriminate the development of multiple primary melanomas (MPM) vs. single site primary melanomas (SPM) using a targeted next generation sequencing panel.

**Materials and Methods:** A total of 39 patients, 20 with SPM and 19 with MPM, were enrolled in our study. Next generation analysis was carried out using a custom targeted sequencing panel that included 32 genes known to have a role in several carcinogenic pathways, such as those involved in DNA repair, pigmentation, regulation of kinases, cell cycle control and senescence.

**Results:** We found a significant correlation between PIK3CA:p.I391M and MPMs, compared to SPMs, *p* = 0.031 and a trend for the association between CYP1B1: p.N453S and SPMs, compared to MPMs (*p* = 0.096). We also found that both subgroups shared a spectrum of 9 alterations in 8 genes (CYP1B1: p.N453S, BAP1: p.C39fs, PIK3CA: p.I391M, CDKAL1: c.1226_1227TG, POLE: p.V1161fs, OCA2: p.R419Q, OCA2: p.R305W, MC1R: p.V60L, MGMT: p.L115F), which suggested that these genes may play a role in melanoma development.

**Conclusions:** In conclusion, despite the small cohort of patients, we found that germline mutations, such as those of PIK3CAand CYP1B1, might contribute to the differential development of SPM and MPM.

## Introduction

Genetic and environmental risk factors contribute to melanoma predisposition. Most genetic alterations underlying this disease occur within melanocytes and an accumulation of genomic changes contributes to melanoma development, progression and evolution (Shain et al., 2015). The presence of heritable germline variants is also an important component of melanoma susceptibility (Potrony et al., 2015).

Genetic and molecular studies on melanoma have led to the identification of some specific alterations in pathways controlling cell proliferation, differentiation and survival (Palmieri et al., 2015). Some specific mutations, called “driver” mutations, promote cancer progression, while many others, known as “passenger” mutations, confer little or no advantage to tumor growth (Reddy et al., 2017). The combination of driver and passenger mutations represents the mutational load of a particular tumor.

Currently, CDKN2A, CDK4, and MC1R have been the most investigated genes involved in melanoma pathogenesis (Potrony et al., 2015). Recent findings have shown how germline variants impact on gene expression, demonstrating that deregulation is an early event in cells “committed” to becoming cancerous (Puig-Butille et al., 2014). To date, the genetic background of single primary melanomas (SPM) and multiple primary melanomas (MPM) has been poorly explored.

According to several studies, the risk of developing an additional primary tumor in patients who have already been diagnosed with primary melanoma ranges from 0.6% to 12.7% (Buljan et al., 2015). It has been reported that individuals with both CDKN2A and MC1R variants have a higher risk of developing MPMs than subjects presenting a CDKN2A mutation but not the MC1R variant (Fargnoli et al., 2010). An association has been also found between MPM and other malignant diseases, suggesting that the latter may share common genetic or environmental factors (Slingluff et al., 1993). However, only few data exist in the literature regarding the genetics of MPM.

The introduction of next generation sequencing (NGS) approaches for the study of cancer cell genomes to determine the mutational landscape of cancers has profoundly enhanced our comprehension of the diseases (Pinto et al., 2014; Serrati et al., 2016).

In the present study, we performed a germline variant analysis in cohorts of patients with SPM and MPM to identify the differentially mutated genes of the two subsets as such an approach could be useful to predict a different predisposition to the two types of melanoma. To this aim, we built a custom targeted NGS panel including 32 genes known to have a role in several carcinogenic pathways, such as those involved in DNA repair, pigmentation, regulation of kinases, cell cycle control, and senescence.

## Materials and Methods

### Patient Information

The study was carried out on 20 patients with SPM and 19 with MPM, all having cutaneous melanoma with no other affected family member. The SPM cases were recruited at the Oncology Unit of the IRCCS Istituto Tumori “Giovanni Paolo II” of Bari, Italy while the MPM cases were enrolled at the Dermatology Department of the University of Bari, Italy.

The study was approved by the local Ethics Committee of the IRCCS Istituto Tumori “Giovanni Paolo II” of Bari (protocol no. 515/EC of May 12, 2015). After obtaining written informed consent, blood samples were collected from all the patients for germline analysis.

### DNA Preparation

DNA was isolated from all blood samples using the QIAamp DNA Blood Midi Kit (Qiagen) according to the manufacturer's instructions. The DNA extracted from each sample was quantified using the Nanodrop and Qubit methods.

### Ion Torrent PGM Library Preparation and Sequencing

Thirty-two genes (CDKN2A, CDK4, BAP1, TERT, TYRP1, MTAP, TYR, NCOA6, MX2, PARP1, ATM, ARNT, CASP8, MC1R, POT1, ACD, TERF2IP, MITF, CYP1B1, SLC45A2, AGR3, CDKAL1, CCND1, BRAF, CTLA4, PIK3CA, MGMT, OCA2, ASIP, XP1, APEX1, POLE) were analyzed by a custom panel (Thermofisher) developed *ad hoc* by our group for germline analyses. Germline BRAF and NRAS gene mutations were tested using a second custom panel previously developed by our group. An input of 10 nanograms/each primer pool was required for both analyses. The tests were conducted as reported in our previous paper (Pinto et al., 2016).

### Variant Calling

Data from the PGM runs were initially processed using the Ion Torrent platform-specific pipeline software, Torrent Suite, to generate sequence reads, trim adapter sequences, and filter and remove poor signal-profile reads. Initial variant calling from the Ion AmpliSeq sequencing data was generated using Torrent Suite Software v5.0 with a plug-in “*variant caller v5.0*” program. In order to eliminate errors in base calling, the Germline High Stringency parameters setting was used to generate the final variant calling. Filtered variants were annotated using the Ion Reporter software v5.0 (Thermo Fisher Scientific). Mutations were visually examined using the Integrative Genomics Viewer (IGV) software (https://www.broadinstitute.org/igv).

Data from the NGS custom panel were also analyzed by a custom pipeline to verify their reliability. Aligned BAM files of samples (TMAP) and Fastq files were downloaded from the Torrent Server. Fastq files were aligned with BWA-mem, obtaining BAM files. Both sets of BAM files were analyzed using the Vardict algorithm (Lai et al., 2016). Variants were then filtered through the vcffilter from the vcflib library. Variants were called when they matched the following conditions: DP > 100 and QUAL > 30. The callset was then intersected with bcftools to include only the variants also called by the TVC VariantCaller plugin.

The selected variants were functionally annotated by Annovar, version 2016Feb01. The LJB^*^ database was used to obtain predictions on deleteriousness from different prediction methods (Dong et al., 2015; Yang and Wang, 2015).

Multiple genomic alteration events were visualized by heatmap using the OncoPrint plot that was designed with the Complex Heatmap R package (Gu et al., 2016).

### Enrichment and Functional Analyses

Webtools GeneMania (Warde-Farley et al., 2010) e gProfiler (Reimand et al., 2007) were used to perform functional and enrichment analyses.

### Statistical Analysis

Statistical analysis was carried out with the SPSS statistical package, version 24 (IBM Inc., Armont, NY, US). Percentage frequency was used to express the characteristics of the population. Categorical variables were compared with the Chi-square test (Fisher's test). A *p* ≤ 0.05 was considered to be significant.

## Results

### Mutational Patterns in SPM and MPM Patients

In the present study, a cohort of patients SPMs (*n* = 20) and MPMs (*n* = 19), sex-matched, was analyzed with our custom NGS panel that included 32 genes involved in melanoma susceptibility (e.g., CDKN2A, BAP1, POT1) and skin/hair pigmentation (e.g., TYR, TYRP1, OCA2).

Interestingly, we found that both subgroups shared a spectrum of alterations in 13 genes (CYP1B1, CASP8, BAP1, PIK3CA, TERT, CDKAL1, CDKN2A, TYR, POLE, OCA2, TERF2IP, MC1R, MGMT), which suggests that they may have role in the development of melanoma. Indeed, 84.61% of the patients carried more than one alteration in the 13 genes listed above. All the patients in the two cohorts were found to be wild type for both BRAF and NRAS.

**Figure 1:**
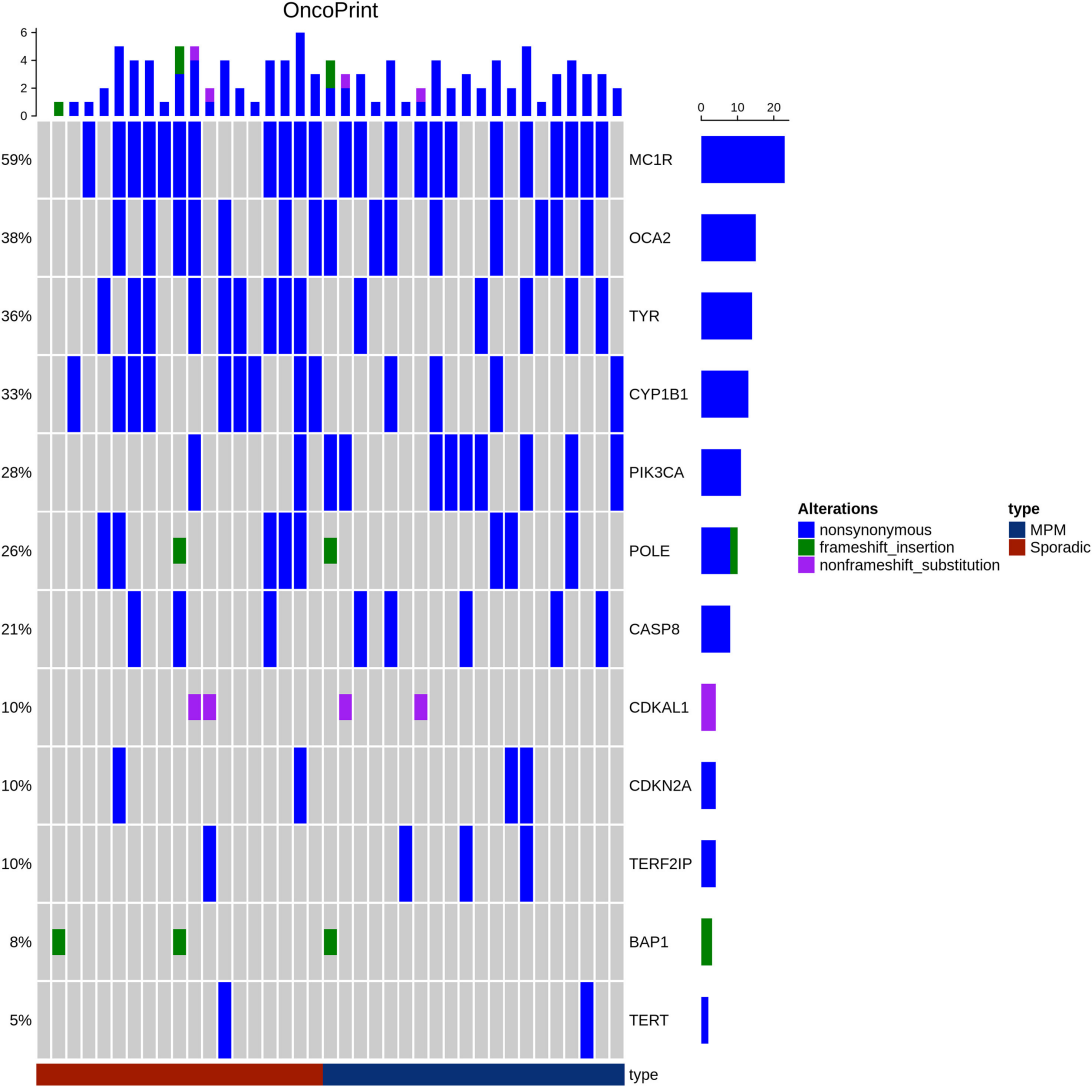
Oncoprint showing alterations in the 13 genes in both patient groups MPM (Blue) and SPM (red). The frequency of each gene alteration in the overall series is reported on the right. The alterations have been reported as non-synonymous SNP (blue), frameshift (green), and non-frameshift (pink) indels. Patients are indicated in the upper line.

An oncoprint of the alterations detected in the 13 genes was drawn (Figure 1). No clear pattern distinguished the MPMs from the SPMs. However, the MC1R variants, in particular p.V60L, were present with almost the same frequency in MPMs and SPMs (60 and 57.89%, respectively). A significant correlation was observed between PIK3CA: p.I391M and the MPMs (45%), as compared to the SPMs (10.52%), *p* = 0.031. Our statistical analysis also revealed a trend for an association between CYP1B1: p.N453S and the SPMs, as compared to the MPMs (47.36% in SPMs vs. 20% in MPMs), *p* = 0.096.

**Figure 2:**
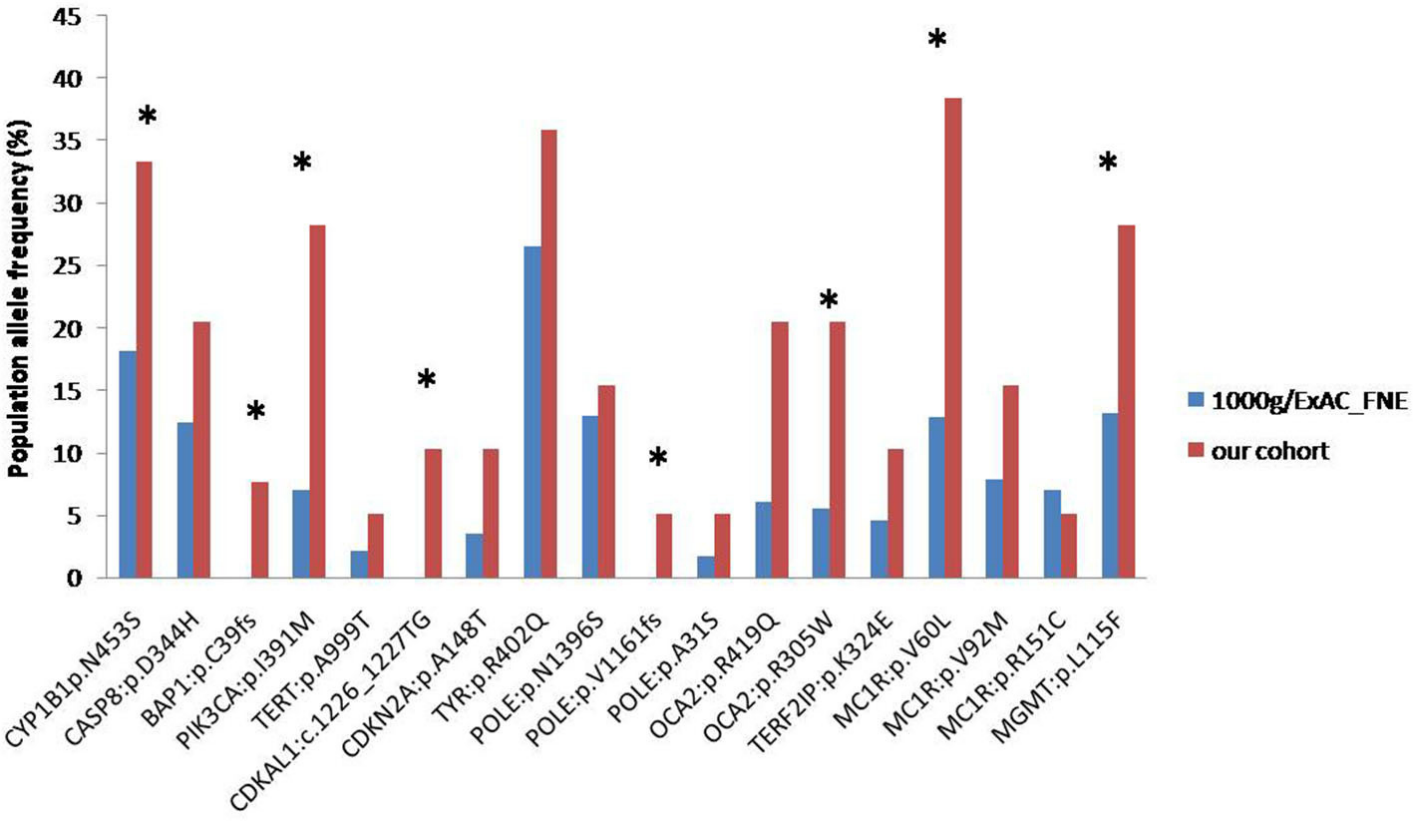
Frequencies of alterations detected in our cohort (red column) and in ExAC/1000 Genomes (blue column) databases (*p < 0.05).

The frequency of alterations observed in our cohort was then compared with the frequency exhibited in the European population from theExAC and 1000 Genomes databases (Figure 2). Nine alterations in eight genes of our cohort showed a statistically significant difference in frequency compared to the population databases (CYP1B1:p.N453S, BAP1: p.C39fs, PIK3CA: p.I391M, CDKAL1: c.1226_1227TG, POLE: p.V1161fs, OCA2: p.R419Q, OCA2: p.R305W, MC1R: p.V60L, MGMT: p.L115F) which suggests they may have a potential role in affecting melanoma susceptibility.

### Enrichment and Functional Analyses

**Figure 3:**
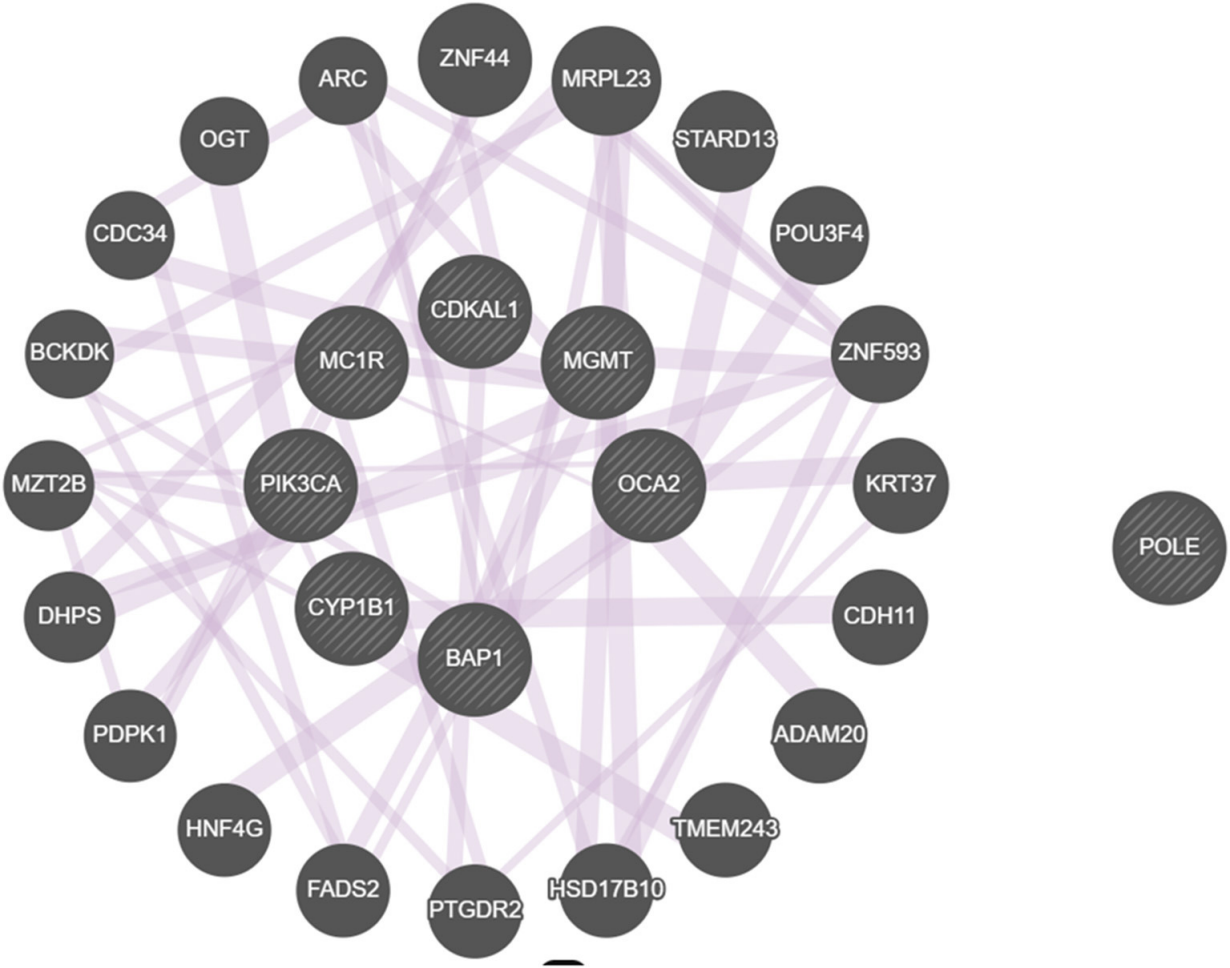
Network built with the GeneMANIA online tool displaying interactions among genes that we found to be mutated. All genes but POLE are connected by co-expression.

We considered the eight genes as a cluster in order to better understand their role in melanomagenesis as a whole. Starting from the list of the eight genes, we built a network through GeneMANIA, a tool which considers physical interactions, co-expression, shared protein domains, pathways, predicted relationships and co-localization data. The eight genes proved to be highly connected especially when their co-expression was considered; only the POLE gene was found not to be connected to any of selected genes (Figure 3).

gProfiler was used to verify if there was a connection amongst the Gene Ontology (GO) terms enriched with our gene list.

Given that we designed a gene panel which is intrinsically enriched for specific pathways, we decided to build an enrichment network of the entire panel through gProfiler and compare it with the network derived from the eight altered genes (Figure 4A). The eight significant mutated genes showed a different enrichment from the entire panel, in particular significant terms “DNA modification” (e.g., alkylation, methylation), “regulation of protein kinase B signaling,” “cellular response to radiation,” and “cofactor binding” were flagged up (Figure 4B).

**Figure 4A:**
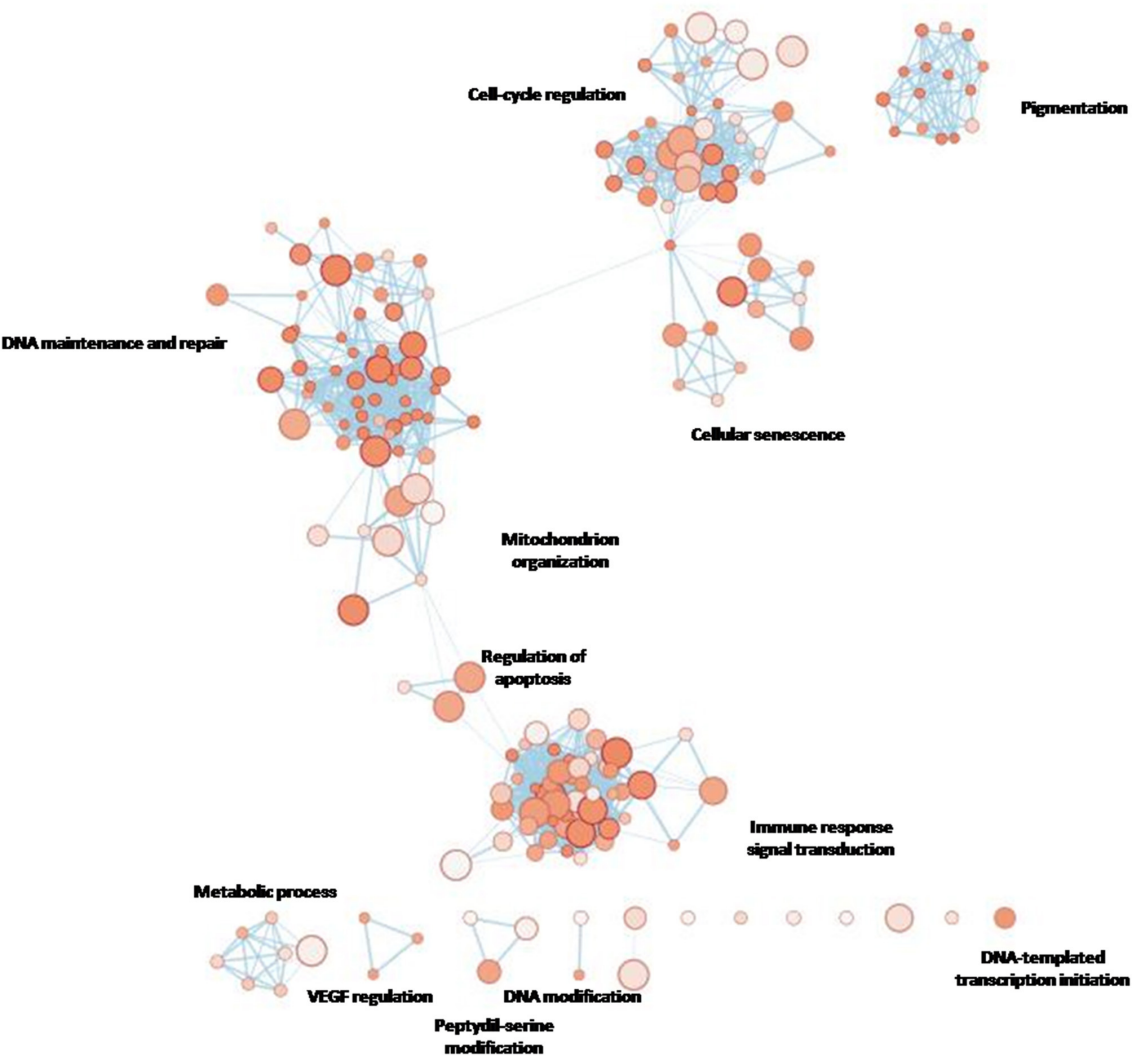
gProfiler network showing the most enriched terms for the entire custom gene panel designed for the present study.

**Figure 4B:**
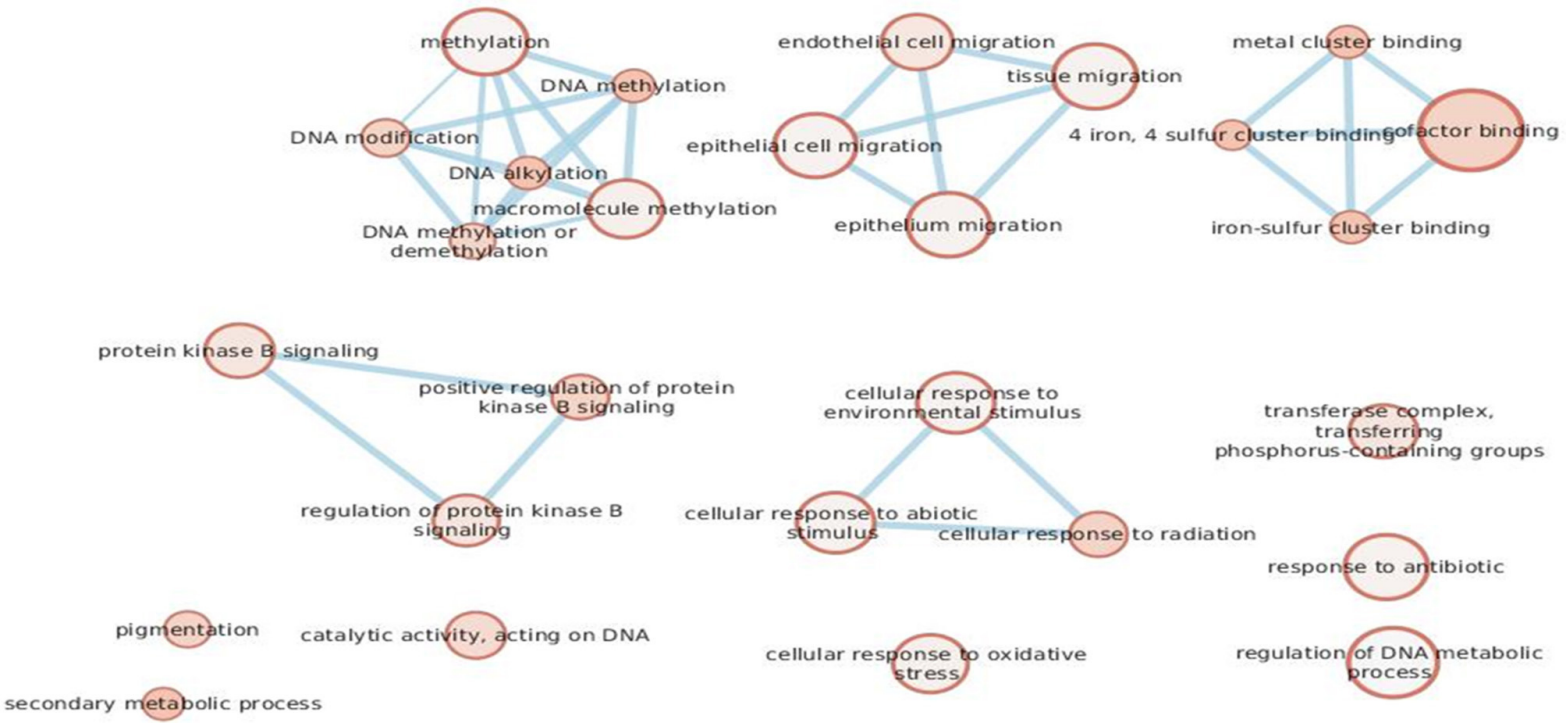
The eight genes found to be mutated in the cohort.

## Discussion

Melanoma is a malignancy with an elevated incidence worldwide. As reported in the Italian Network of Cancer Registries' (AIRTUM) annual report (Coviello et al., 2017), melanoma is the tumor with the third highest incidence in young people and both genders in Italy. The disease has a heterogeneous etiology due to different genetic and environmental predisposing factors: 5–10% of melanomas occur in patients with a family history of the disease (Florell et al., 2005) and multiple primary melanoma cases may present an inherited susceptibility (Puig et al., 2005). However, to date, only 30% of susceptibility to melanoma in general population can be explained by the presence of risk genes. Gene-environment interaction in families may account for different alteration patterns also in low/moderate risk genes in high-risk families (Potrony et al., 2015).

Given the limited literature regarding the genetics of SPM and MPM, we performed an exploratory analysis in a selected cohort of 19 patients with SPM and 20 with MPM to identify alterations which may discriminate between the two groups. We specifically investigated genes known to have a role in pathways implicated in carcinogenesis, such as those involved in DNA repair, pigmentation, kinases function regulation, cell cycle control, and senescence.

In a previous paper, we analyzed the coexistence of MPM and oculocutaneous albinism (De Summa et al., 2017) and concluded that MGMT is a new player involved in melanoma pathogenesis. In this study, we found that MC1R variants were almost equally distributed across both groups of patients, those with SPMs and those with MPMs. The correlation between MC1R variants and melanoma risk has been widely described in different populations (Tagliabue et al., 2015; Pinto et al., 2016; Pellegrini et al., 2019). The MC1R gene is considered a master regulator of pigment production and distribution throughout the skin (pigmentary function) (Puig et al., 2005) as well as a regulator of antioxidant defenses and DNA repair mechanisms (non-pigmentary function) (Rees, 2004; Abdel-Malek et al., 2014), thereby supporting its role in melanoma development. In our study, V60L was the most common MC1R variant in both groups, a finding which is in agreement with the data regarding the distribution of this variant in patients from the South-East of Italy reported by Guida et al. (2015) and Garcia-Borron et al. (2014).

Interestingly, we found a significant association between PIK3CA_I391M and MPMs and a trend for an association between CYP1B1_N453Sand SPMs, both of which had not been reported in previous studies.

Mutations in the gene encoding the catalytic subunit of PI3K, PIK3CA, occur at very low frequencies (<5%) in melanoma (Guida et al., 2015), although they are very frequent in other human cancers (Omholt et al., 2006). Specifically, the I391M mutation of PIK3CA has been reported in prostate cancer (Lai et al., 2015), breast cancer (Lo Iacono et al., 2016) and melanoma (Pinto et al., 2016). In a previous paper (Pinto et al., 2016), we performed an *in silico* analysis that pointed to the harmful potential of this mutation in PIK3CA activity, leading to constitutive AKT activation and dysregulation of proliferation, metabolism, and protein synthesis, as well as angiogenesis and apoptosis (Omholt et al., 2006).

CYPB1 encodes a Cytochrome P450 (CYP) enzyme that is mainly involved in drug metabolism (Soysal et al., 2019). The role of CYPB1 in cancers has been explored and CYPB1 was shown to be widely expressed in human malignancies, but silent in most normal tissues (Agundez, 2004). Mutations in codon 453 have not exhibited any clear association with altered enzyme activity (Soysal et al., 2019). However, the N453S mutation has been associated with increased formation of catechol estrogens (Murray et al., 2001). Catechols have a proven cytotoxic activity in cells, thereby contributing to estrogen-induced carcinogenesis (such as in the breast) (Gaudet et al., 2006).

Melanoma has been widely investigated as a steroid hormone-sensitive cancer in the last 30 years and many epidemiological studies have explored the relationship between estrogens and melanoma, without providing any definitive result to date (Shen et al., 1997).

While the contribution of somatic BRAF/NRAS mutations to melanoma is very important, our study did not reveal any evidence of germinal BRAF/NRAS mutations. Other interesting results from our study regarded the alterations shared by both groups. A total of eight mutated genes (CYP1B1, BAP1, PIK3CA, CDKAL1, POLE, OCA2, MC1R, MGMT), distributed between the MPM and the SPM cases, were identified as having allelic frequencies that were significantly higher than those of the general population reported in ExAC database and the 1000 Genomes Project. We decided to consider the eight mutated genes as a cluster to perform pathway enrichment analysis: the terms “DNA modification” (e.g., alkylation, methylation), “regulation of protein kinase B signaling,” “cellular response to radiation,” and “cofactor binding” were the most significant.

It is now clear that aberrant regulation of DNA methylation plays an important role in the development and progression of cancer, cutaneous malignant melanoma (CMM) included. The malignant transformation of healthy melanocytes requires not only genetic changes but also epigenetic alterations. Enzymes establishing DNA methylation patterns, such as DNA methyltransferases DNMT3A and DNMT3B, are significantly upregulated during CMM progression (Dika et al., 2019). Aberrant promoter DNA hypermethylation preferably occurs at CpG dinucleotide regions, also known as CpG islands, resulting in the downregulation of tumor suppressor gene expression. Several studies on melanoma cases have reported that more than 70 genes involved in fundamental pathways such as those of cell cycle regulation, DNA repair, apoptosis, cancer invasion, metastasis and growth, are hypermethylated (Nguyen et al., 2011).

Moreover, in CMM, DNA methylation loss (hypomethylation) leads to activation of normally silenced cancer germline genes, such as the melanoma antigen (MAGE) genes, contributing to progression of the malignancy (van den Hurk et al., 2012). In another study, although methylation levels for most investigated gene promoters were very low, Hyland and coworkers observed a significantly reduced promoter methylation and increased expression (fold change = 1.27, *P* = 0.048) for TNFRSF10C in the blood PBMCs of the cases they examined and thus concluded that this gene may have a role in melanoma susceptibility (Sigalotti et al., 2002).

As could be observed in the network of GO terms enriched with our selected genes, “cellular response to radiation” showed significance. The role of UV damage in melanomagenesis is well-known. Indeed, in our cohort, MC1R:p.V60L was the most frequent alteration and it has been shown elsewhere to be responsible for a low eumelanin/pheomelanin ratio that is a risk factor for skin cancer (Hyland et al., 2014).

Recently, two research groups studied the germline genome of more than 20 types of cancer from several consortia (Scott et al., 2002; Huang et al., 2018). Although a fraction of malignancies are known to be due to more than 100 genes that harbor predisposing alleles, previous studies focused on a single cancer type while emerging evidence has demonstrated that predisposing factors span across multiple cancers. Scott et al. (2002) and Huang et al. (2018) showed that many pathogenic germline variants are present in cancer patients and, interestingly, they demonstrated a link between the germline and the somatic genome. As an example, an analysis of TCGA data indicated that a specific haplotype on chromosome 15q22.2 was associated with an increased GNAQ copy number. Guida et al. (2015) showed that a germline haplotype at locus 19p13.3 was associated with altered regulation of the PIK3CA/mTOR pathway. The results of these studies, together with the GO term “regulation of protein kinase B” observed in our cohort, highlight the role of predisposing factors across cancer types and may help us better understand the pattern of shared germline alterations. In sum, melanomagenesis may be explained through the term “heritability”—which refers to the variance of a phenotype due to genetic and environmental effects—since the interaction between genes (eight mutated genes) and important environmental factors (such as sun exposure) could exponentially increase the risk of developing the disease.

In conclusion, the biological implications of our results are linked to the statistical significance of the alteration frequencies seen in our cohort compared to those of the general population databases (ExAC and 1000 Genomes) in that they showed a greater number of alterations that were meaningful for melanoma susceptibility. Furthermore, despite the small cohort of patients, our findings suggest that certain germline mutations, such as those of PIK3CA and CYP1B1, may contribute to the differential development of SPM and MPM. We will perform further studies that will also examine somatic alterations in larger cohorts of patients to gain additional insights into the pathogenesis of SPM and MPM.

Further studies will be performed implementing the set of patients, including the analysis of the somatic alterations, in order to gain new insights into the pathogenesis of SPMs and MPMs.

## Data Availability Statement

The datasets presented in this study can be found in online repositories. The names of the repository/repositories and accession number(s) can be found at: NCBI BioProject, https://www.ncbi.nlm.nih.gov/Traces/study/?acc=PRJNA679171, accession: PRJNA679171.

## Ethics Statement

The studies involving human participants were reviewed and approved by Ethics Committee of the IRCCS Istituto Tumori Giovanni Paolo II of Bari (Protocol No. 515/EC of May 12, 2015). The patients/participants provided their written informed consent to participate in this study.

## Author Contributions

SDS and SG wrote the paper and did the statistical analyses. SDS did the bioinformatics. AL, RP, SS, RE, and EN did the wet lab and clinical work. GGi, GGu, and MG provided patient enrollment and selection. AA, GGu, ST, and NS contributed in discussing the results and wrote the paper.

## Conflict of Interest

The authors declare that the research was conducted in the absence of any commercial or financial relationships that could be construed as a potential conflict of interest.

## Abbreviations

SPM: single primary melanoma
MPM: multiple primary melanoma.
